# Green Chemical Shear-Thickening Polishing of Monocrystalline Silicon

**DOI:** 10.3390/nano14231866

**Published:** 2024-11-21

**Authors:** Jiancheng Xie, Feng Shi, Shanshan Wang, Xing Peng, Qun Hao

**Affiliations:** 1 School of Optics and Photonics, Beijing Institute of Technology, Beijing 100081, China; 2 National Key Laboratory on Near-Surface Detection, Beijing 100072, China; 3 College of Intelligence Science, National University of Defense Technology, Changsha 410073, China; pengxing22@nudt.edu.cn; 4 School of Opto-Electronic Engineering, Changchun University of Science and Technology, Changchun 130022, China

**Keywords:** green chemical shear-thickening polishing, monocrystalline silicon, material removal, low damage

## Abstract

A green chemical shear-thickening polishing (GC-STP) method was studied to improve the surface precision and processing efficiency of monocrystalline silicon. A novel green shear-thickening polishing slurry composed of silica nanoparticles, alumina abrasive, sorbitol, plant ash, polyethylene glycol, and deionized water was formulated. The monocrystalline silicon was roughly ground using a diamond polishing slurry and then the GC-STP process. The material removal rate (MRR) during GC-STP was 4.568 μmh^−1^. The material removal mechanism during the processing of monocrystalline silicon via GC-STP was studied using elemental energy spectroscopy and FTIR spectroscopy. After 4 h of the GC-STP process, the surface roughness (Ra) of the monocrystalline silicon wafer was reduced to 0.278 nm, and an excellent monocrystalline silicon surface quality was obtained. This study shows that GC-STP is a green, efficient, and low-damage polishing method for monocrystalline silicon.

## 1. Introduction

Monocrystalline silicon (Si) is the predominant semiconductor material utilized in the electronics industry, exhibiting a band gap of 1.12 eV (at 26 °C) and a considerable range of doping resistivity [1]. Silicon wafers with superior surface quality are essential for improving the performance of silicon-based semiconductor components. Silicon slicing, grinding, and polishing have all been successfully applied for the large-scale processing of silicon-based devices [2]. The surface quality of silicon wafers is significantly influenced by the polishing process. Consequently, a large number of studies have focused on the process of polishing silicon wafers to improve their surface quality [3].

Currently, ultra-precision processing technologies represented by chemical mechanical polishing (CMP), magnetorheological polishing (MFR), and ion-beam sputtering removal are utilized for the ultra-smooth polishing of wafer surfaces. However, it remains difficult for magnetorheological polishing and ion-beam sputtering removal processes to be applied in the mass production of wafers due to the complexity and high cost of these processes [4]. The surface roughness after chemical–mechanical polishing can usually reach about 1 nm. After substantial research and improvement, chemical mechanical polishing (CMP) has been widely applied to the fabrication of silicon wafers to reduce the subsurface damage generated during the monocrystalline silicon fabrication process and to improve the surface quality [5,6].

The chemical mechanical polishing (CMP) mechanism involves chemical modification of the wafer surface using a chemical–mechanical polishing solution, and then the modified layer is removed by abrasive machinery [7]. The MRR of CMP can be adjusted using Preston’s equation, which states that the polishing speed is affected by the distribution of the polishing pressure, the properties of the polishing plate, and the relative velocities of the abrasive and workpiece [8]. Wang et al. investigated the effect of chemical additives on the material removal of monocrystalline silicon [9]. S. Lu et al. improved the polishing slurry by adding isopropyl alcohol chemical for improving the quality of monocrystalline silicon surfaces [10]. K. Yang et al. evaluated the effect of abrasive grains and polishing pads on the removal rate and surface quality of monocrystalline silicon materials. W. Xie et al. developed a new polishing fluid to study the effect of polishing pressure on the surface quality of monocrystalline silicon [11]. Currently, some improved processes for chemical–mechanical polishing are proposed for the processing of semiconductors or optical components. Chemical–mechanical polishing processes are able to reduce the roughness of a monocrystalline silicon surface to less than 1 nm [12]. R. Pan et al. improved the material removal rate (MRR) during polishing by modifying the polishing tool and optimizing the removal function model [13]. Y. Xu et al. developed a gel-flexible polishing tool for chemical mechanical polishing to improve the surface quality and processing efficiency of sapphire wafers [14]. Z. Wang et al. combined chemical mechanical polishing with air-jet polishing to improve the surface of optical glass [15]. A. Lu et al. proposed a biaxial wheel polishing technique and a mathematical model for predicting the surface morphology and roughness [16]. X. Yang et al. proposed electric field-assisted chemical–mechanical polishing to achieve ultra-precision polishing of silicon carbide with a material removal rate as high as 23 μmh^−1^ [17]. However, the traditional chemical–mechanical polishing slurry contains a large number of unfriendly chemical additives, such as inorganic acids, inorganic bases, complexing agents, etc., and is therefore not environmentally friendly.

Currently, non-Newtonian fluids are being used in ultra-precision machining as a special new type of slurry. Crawford [18] et al. found that during CMP, the shear-thickening performance of the STF under high-shear conditions will affect the surface integrity of the workpiece after polishing, and can well avoid the inhomogeneity caused by scraping the workpiece surface. However, the surface quality of the shear-thickening polishing (STP) process is weakened by the lack of a chemical mechanism. Li et al. [19,20,21] proposed shear-thickening polishing using non-Newtonian fluid properties and successfully achieved the “flexible polishing” of workpieces using the shear-thickening mechanism of specific abrasives.

In this paper, a green chemical shear-thickening polishing (GC-STP) method for the ultra-precision polishing of monocrystalline silicon is proposed. In our work, a new concept of green chemical shear-thickening polishing slurry (GC-STPS) is proposed. It mainly consists of deionized water, polymer (polyethylene glycol), dispersant (nano-silica particle), abrasive (Al_2_O_3_), plant ash (K_2_CO_3_), and sorbitol. After green chemical shear-thickening polishing, the monocrystalline silicon surface was cleaned of any impurities via ultrasonic vibration. The surface roughness of the polished monocrystalline silicon was tested to be Ra = 0.278 nm, which is commercially significant (Ra = 0.5 nm). The mechanism of green shear-thickening polishing was explained through scanning electron microscope electron elemental energy analysis (EDS) and Fourier transform infrared spectroscopy (FTIR).

## 2. Experiments

In this study, a 99% purity monocrystalline silicon wafer (China TDG Holdings Co., Ltd., Haining, China) was used for related experiments, which had a length of 20 mm, a width of 20 mm, and a thickness of 1 mm. The initial surface roughness (Ra) and PV values of the monocrystalline silicon wafer were 18 nm and 743 nm. A precision polishing machine (ZYP230, Shenyang Mike Material Processing Equipment Co., Ltd., Shenyang, China) was used for the GC-STP.

Polyethylene glycol (PEG) is a polymer with good adhesive properties that is non-toxic, non-irritant, and widely applied in various pharmaceutical preparations. In this study, PEG-600 was used as a shear-thickening phase for the polishing slurry. Plant ash is the residue of burning plants (herbaceous and woody plants), and is non-toxic and alkaline in an aqueous solution. Sorbitol is a food additive that is widely distributed in fruits and also non-toxic. It is a natural chemical complexing agent. Nano-silica is a non-toxic, non-polluting, and water-insoluble nanoparticle. In this study, 400 nm silica nano-silica was employed as the dispersant for the new slurry.

**Figure 1:**
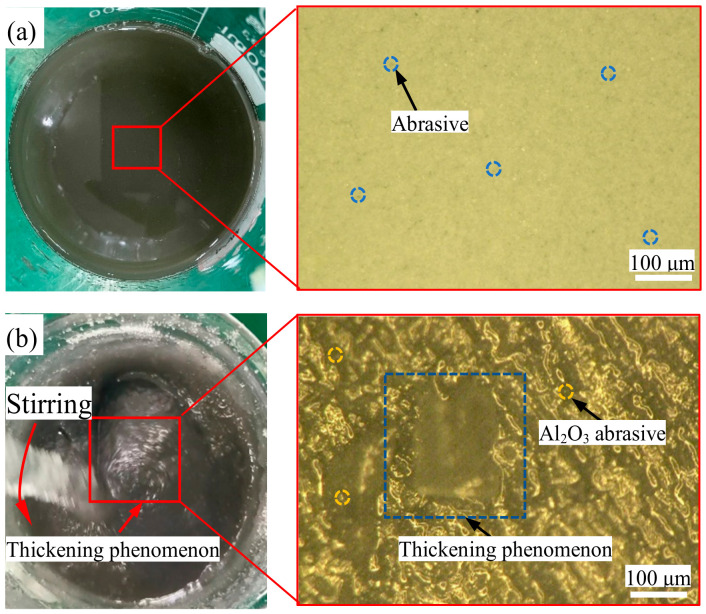
Schematic diagram of green chemical shear-thickening polishing slurry (GC-STPS): (**a**) non-disturbed state; (**b**) disturbed state.

Green chemistry shear-thickening and polishing slurry (GC-STPS) consists of a shear-thickening base fluid, an abrasive, and a green chemical additive. In this study, the specific preparation process was as follows: First, deionized water and polyethylene glycol were mixed in equal proportions to make a shear-thickening base fluid. Secondly, green chemical additives (plant ash, sorbitol) were added to the shear-thickening base fluid. Then, the dispersant (nano-silica) and an Al_2_O_3_ abrasive with a particle size of 1 μm were added to the liquid and stirred well, and ultrasonic dispersion was carried out for 30 min to break the aggregation in order to keep the fluid homogeneous. The prepared green chemical shear-thickening polishing slurry had a pH of 10. Without external disturbances, the components of the green chemical shear-thickening polishing slurry were uniformly distributed in the slurry, as shown in Figure 1a. When the polishing slurry was disturbed, its viscosity changed dramatically, and the GC-STPS became thickened, as shown in Figure 1b.

Before carrying out the experiment, the surface of the monocrystalline silicon was tested with a scanning electron microscope and the surface roughness was tested using a white-light surface profiler. The quality of the monocrystalline silicon wafers before and after the experiment was measured with an electronic balance. After GC-STP, the surface roughness of the monocrystalline silicon wafers was measured with a scanning electron microscope (SEM), an atomic force microscope (AFM), and a ZYGO interferometer. Transmission electron microscopy (JEOLJEM-2100FTEM, 200 kV, Thermo Fisher Scientific, Waltham, MA, USA) was used to characterize the nano-silica and alumina abrasives, and a particle size analyzer (Silicon Nano ZS90, Malvern Analysis Co., Ltd., Malvern, UK) was used to measure the diameter distribution of the nano-silica and Al_2_O_3_ abrasives in GC-STPS. Scanning electron microscope elemental energy-dispersive analysis (EDS) (SEM type: Hitachi SU3500, Hitachi, Tokyo, Japan) was used to conduct elemental analyses and study the surface of the monocrystalline silicon wafers after GC-STPS. An FTIR spectrometer was used to measure the vibration peak changes after sorbitol chelation.

## 3. Results

**Figure 2:**
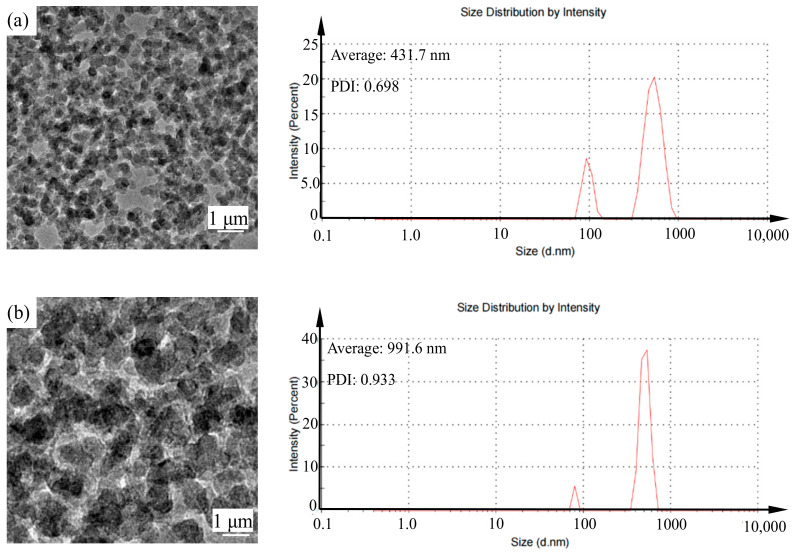
TEM images of (**a**) nano-silica in GC-STPS; (**b**) Al_2_O_3_ abrasive in GC-STPS.

The configured new green chemical shear-thickening polishing slurry contains a nano-silica particle dispersant and an alumina abrasive. As shown in Figure 2a, the nano-silica particles and alumina abrasives are each uniformly distributed in the non-Newtonian fluid polishing slurry. The diameter of the nano-silica particles is 431 nm, and the diameter of the alumina abrasive is 991.6 nm (Figure 2b). There is no agglomeration of silica nanospheres in the colloidal silica and non-Newtonian fluid polishing slurry, which is beneficial for achieving excellent polishing performance. The homogeneity and distribution properties of alumina abrasives are better than those of other previously reported polishing slurries.

**Figure 3:**
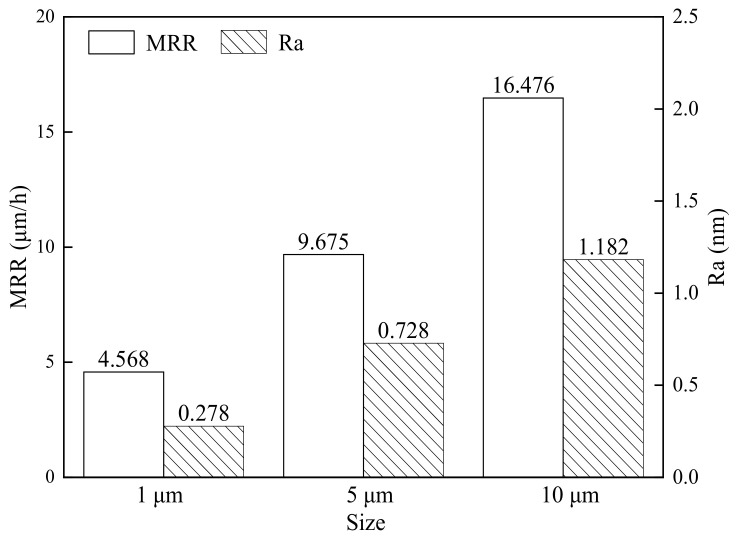
The influence of Al_2_O_3_ abrasive diameter on MRR and surface roughness (R_a_) of monocrystalline silicon wafers.

Figure 3 depicts the effect of the Al_2_O_3_ abrasive diameter on the material removal rate (MRR) and surface roughness (Ra) of GC-STP-polished monocrystalline silicon. Al_2_O_3_ abrasives with diameters of 1 μm, 5 μm, and 10 μm were added to the GC-STPS for monocrystalline silicon GC-STP, and the resulting material removal rates were 4.568 μmh^−1^, 9.657 μmh^−1^, and 16.476 μmh^−1^, with surface roughness (Ra) values of 0.278 nm, 0.728 nm, and 1.182 nm. From the figure it can be seen that the MRR and Ra are positively correlated. Before GC-STP, the monocrystalline silicon wafers were ground and roughly polished using 3 μm polycrystalline diamond and 300 nm alumina abrasives. During the grinding and rough-polishing process, the MRR value is very high and the surface roughness increases significantly (i.e., the surface quality deteriorates). For the GC-STP of monocrystalline silicon, the material removal rate and surface quality can be balanced by combining grinding, rough-polishing, and GC-STP with abrasives of different diameters.

**Figure 4:**
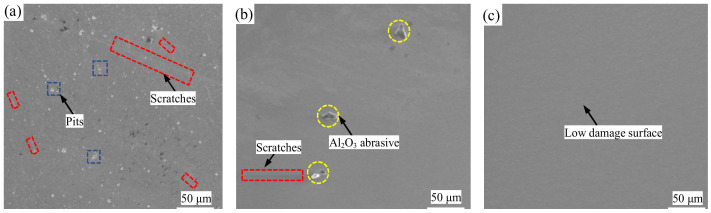
SEM image of monocrystalline silicon wafer surface after GC-STP: (**a**) before polishing; (**b**) after 2 h of GC-STP; (**c**) after 4 h of GC-STP.

Figure 4 shows SEM images of the monocrystalline silicon wafers before polishing, after 2 h of GC-STP, and after 4 h of GC-STP. Before polishing, there were scratches on the surface of the monocrystalline silicon wafers (Figure 4a). After 2 h of GC-STP, the scratches on the surface of the monocrystalline silicon wafers had become distributed (Figure 4b). After 4 h of STP and ultrasonic cleaning of the surface, the scratches and pits on the surface were almost invisible and the polished monocrystalline silicon surface was smooth, with fewer defects and no remaining surface nanoparticles (Figure 4c). Therefore, the green chemical shear-thickening polishing (GC-STP) proposed in this study is a highly efficient and ultra-low-damage polishing technique for monocrystalline silicon.

**Figure 5:**
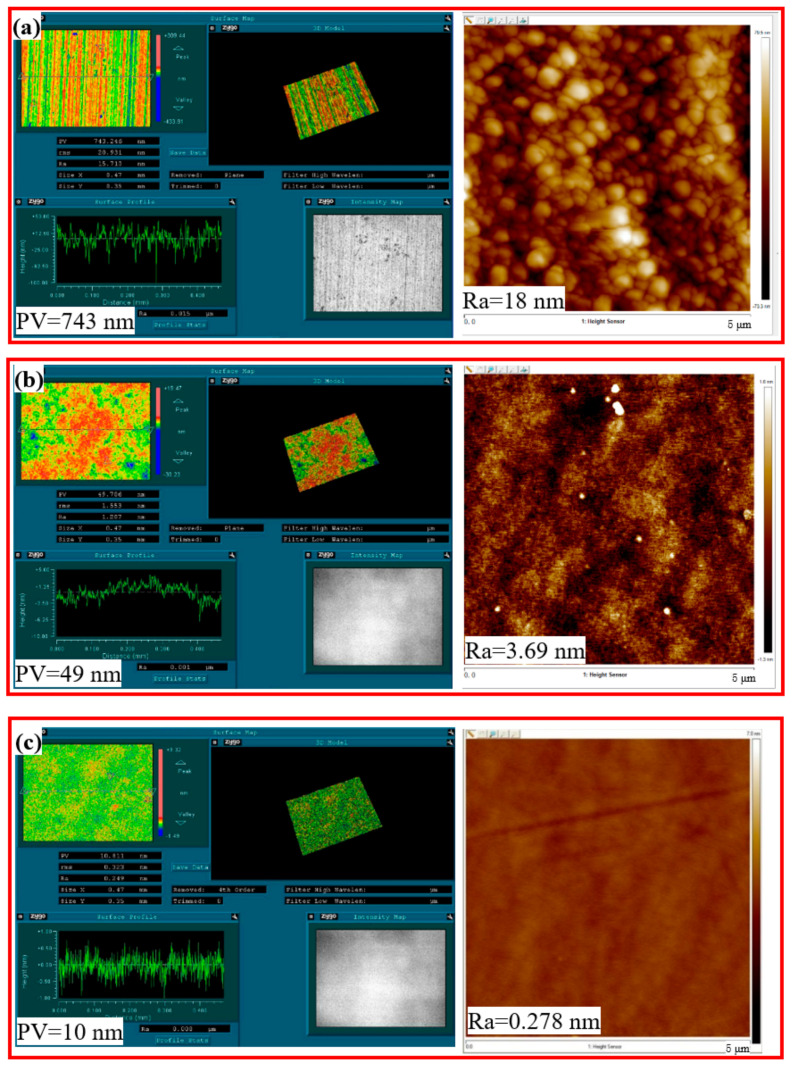
Scanning electron microscope image of monocrystalline silicon face shape accuracy and roughness after GC-STP: (**a**) before polishing; (**b**) after 2 h of GC-STP; (**c**) after 4 h of GC-STP.

Furthermore, a white-light interferometer and an atomic force microscope were used to detect the monocrystalline silicon surface shape accuracy and surface roughness; the initial roughness was measured as Ra = 246 nm and PV = 483 nm, as shown in Figure 5a. After 2 h of GC-STP, the monocrystalline silicon surface shape accuracy and roughness had become optimized. In this case, Ra = 3.69 and PV = 49 nm, as shown in Figure 5b. After 4 h of GC-STP, the surface quality of the monocrystalline silicon was significantly enhanced: the surface roughness (Ra) was reduced to 0.278 nm and the PV was reduced to 10 nm. Thus, the green chemical shear-thickening polishing (GC-STP) method proposed in this study is a highly efficient and ultra-low-damage technique for polishing monocrystalline silicon.

## 4. Discussion

The surface roughness of monocrystalline silicon was reduced from 18 nm to 0.278 nm by GC-STP. Al_2_O_3_ abrasives was applied for GC-STP. The hardness of the abrasives, in order, is as follows: diamond (10 Mohs) > Al_2_O_3_ (9 Mohs) > SiO_2_ (7 Mohs). These three abrasives can quickly remove scratches and defects from monocrystalline silicon surfaces. Silica nanoparticles and Al_2_O_3_ abrasives exhibit remarkable homogeneity and dispersion in colloidal GC-STP slurries. In this study, we were able to avoid the nanoparticles aggregating on the surface of the monocrystalline silicon to cause scratches. Therefore, the green chemical shear-thickening polishing (GC-STP) technique applied in this study is a highly efficient and ultra-low-damage single-crystal silicon processing technique.

In order to measure and characterize the surface of the monocrystalline silicon after GC-STP, the monocrystalline silicon was cleaned with ultrasonic vibration. The molecular structure of plant ash, which is the non-toxic and non-polluting ash byproduct of herb burning, is K_2_CO_3_, and its aqueous solution is alkaline. Sorbitol occurs extensively in fruits such as pears, peaches, and apples and is a natural food additive. Aluminum dioxide is found in sand, crystals, and stones in nature. Our GC-STPS contains Al_2_O_3_, sorbitol, plant ash, and deionized water. Therefore, the polishing liquids and processes used in this study are eco-friendly and “green” [22].

**Figure 6:**
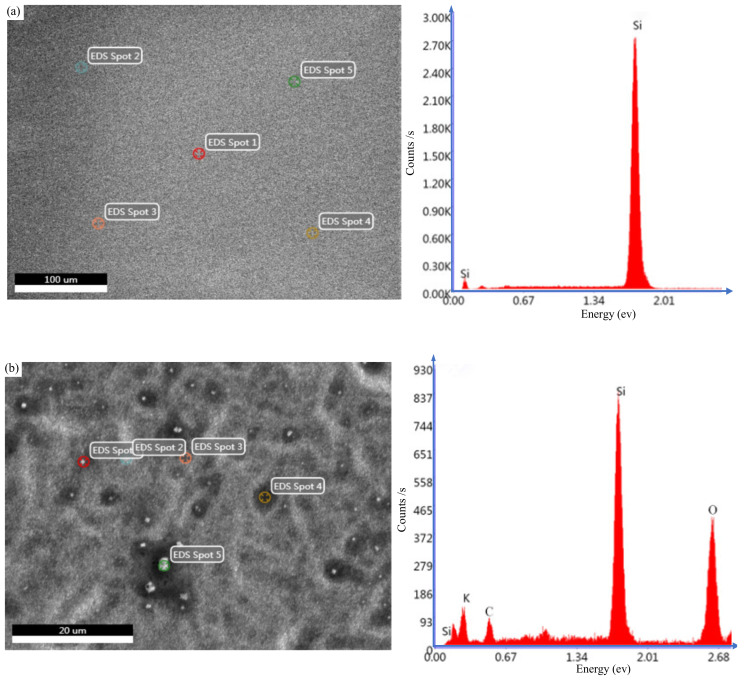
Energy spectra of elements on the surface of monocrystalline silicon: (**a**) original surface elements; (**b**) surface elements of monocrystalline silicon after immersion in GC-STPS.

In order to explore the mechanism of GC-STP, we analyzed the elements on the original surface of monocrystalline silicon and the surface elements of monocrystalline silicon after immersion in GC-STPS, as shown in Figure 6. The main element observed on the pristine surface was silicon (Si), as shown in Figure 6a. After 2 h of GC-STP, oxygen (O) and potassium (K) were observed on the surface of the monocrystalline silicon wafer because the plant ash (K_2_CO_3_) in the GC-STPS had chelated with the surface of the monocrystalline silicon to form an oxide layer, as shown in Figure 6b. Theoretically, the oxidation reaction of monocrystalline silicon first produces Si(OH)_2_^2+^, and the formed Si(OH)_2_^2+^ then further undergoes chelation to form Si(OH)_4_; however, Si(OH)_4_ is unstable and easily decomposes to form oxide SiO_2_(OH)_2_^2−^. Loose oxides are easily removed due to the micro-shearing/cutting action of “flexible abrasives” [19,23]. Therefore, the increase in the surface oxide content of monocrystalline silicon has a positive effect on improving the processing efficiency of the GC-STP process. After 1 h of polishing, specific layers on top of the scratches and grooves were completely removed, forming a microchip. However, due to the small chemical contact area and high shear resistance, scratches and loose modified layers at the bottom of the grooves will only produce chips after a longer period of polishing. According to the elemental analysis and theoretical analysis spectrum, the following reaction equations are proposed for the GC-STP process [24]:

(1) $${\mathrm{Si}+2\mathrm{OH}}^{-}\;\to {\mathrm{Si}(\mathrm{OH})}_{2}^{2+}\;$$

(2) $${\mathrm{Si}(\mathrm{OH})}_{2}^{2+}{+2\mathrm{OH}}^{-}\;\to {\mathrm{Si}(\mathrm{OH})}_{4}\;$$

(3) $${\mathrm{Si}(\mathrm{OH})}_{4}\;\to {\mathrm{SiO}}_{2}{\left(\mathrm{OH}\right)}_{2}^{2-}+2H^{+}$$

**Figure 7:**
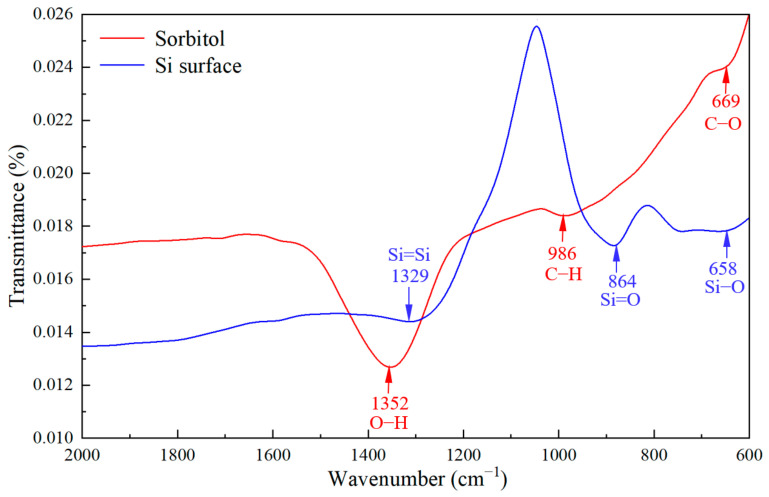
FTIR spectra of sorbitol and monocrystalline silicon surface chelation.

**Figure 8:**
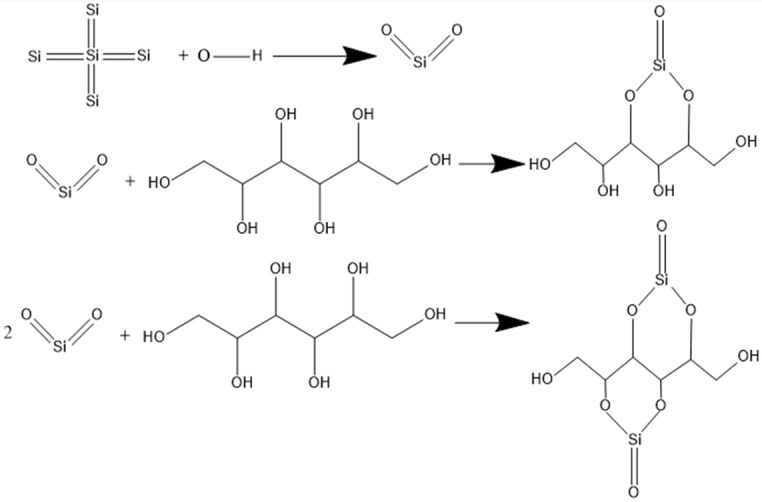
Chelating equation for the GC-STP of monocrystalline silicon.

In order to verify the chelation of sorbitol with Si(OH)_2_^2+^ ions, we compared the FTIR spectra of GC-STP-processed monocrystalline silicon surfaces as shown in Figure 7. The peaks at the center of 1352 and 1329 cm^−1^ represent the O-H stretching vibration and Si=Si bending vibration. The peaks at 986 and 864 cm^−1^ are the C-H and Si=O stretching vibrations. The peaks at 669 and 658 cm^−1^ correspond to the bending and torsion vibrations of C-O and Si-O. The chelation of sorbitol and Si(OH)_2_^2+^ ions results in a shift of the peak to lower wavenumbers. From the above analyses, we know that the monocrystalline silicon in GC-STP chemically reacts with the GC-STPS. The chemical reaction formula is shown in Figure 8.

**Figure 9:**
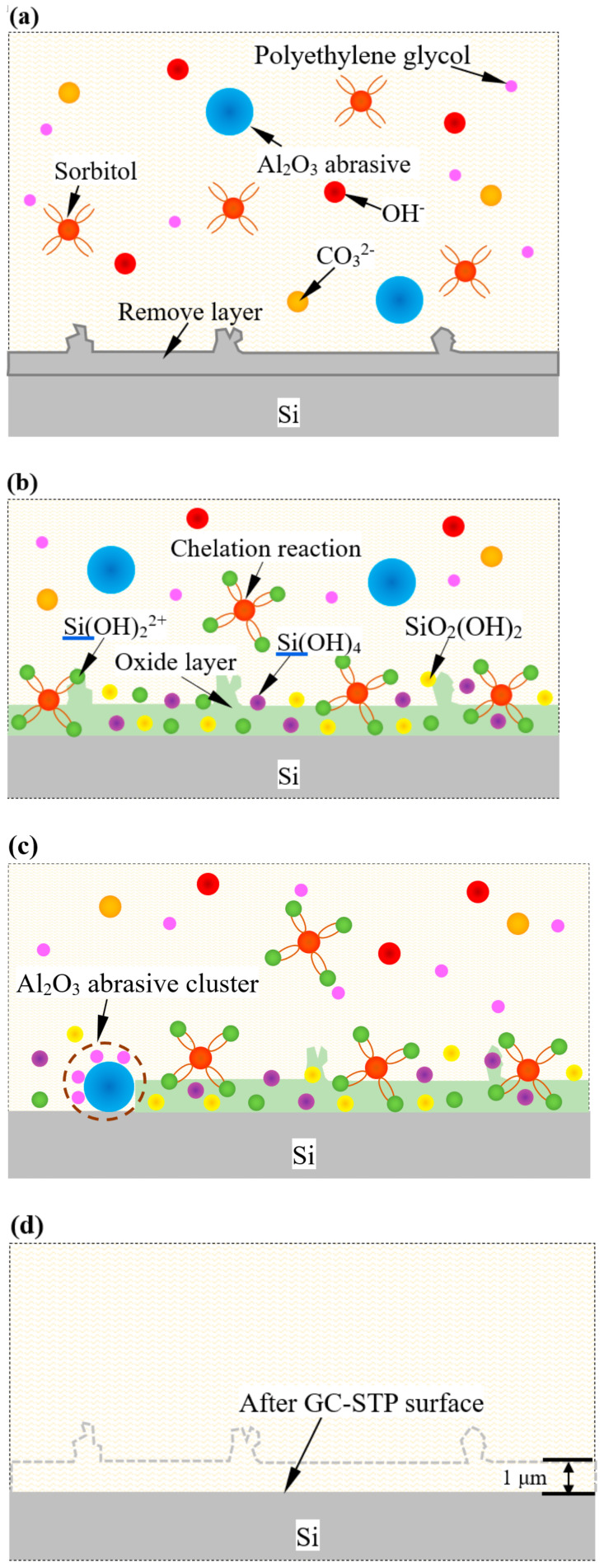
Diagrammatic representation of the monocrystalline silicon GC-STP mechanism: (**a**) chemical softening; (**b**) complexation reaction in GC-STP; (**c**) removal of the softened layer by the abrasive; (**d**) ultra-smooth surface of monocrystalline silicon formed after GC-STP.

The mechanism of GC- STP-processed monocrystalline silicon was revealed by applying XPS and FTIR spectroscopy, as shown in Figure 9. Grass ash releases hydroxyl ions to form an alkaline environment in the GC-STPS (Figure 9a). Under these alkaline conditions, Si(OH)2^2+^ intermediates are gradually formed on the surface of the monocrystalline silicon, and sorbitol and Si(OH)_2_^2+^ intermediates further react chemically to form tetrahydroxyl-coordinated Si(OH)_4_ compounds. Under stress conditions, Si(OH)_4_ compounds are unstable and decompose into SiO2(OH)_2_^2−^. Therefore, a softened layer is formed by the Si(OH)_2_^2+^ ions, Si(OH)_4_ compound intermediates, and SiO_2_(OH)_2_^2−^, as shown in Figure 9b. In the GC-STP process, the GC-STPS thickens rapidly under the action of shear force, and the polyethylene glycol (PEG-600) in the polishing slurry wraps the Al_2_O_3_ abrasive to form Al_2_O_3_ abrasive clusters in order to stably remove the softening layer, as shown in Figure 9c. According to abrasive cutting theory, the depth of material removal in the GC-STP process is mainly related to the Al_2_O_3_ abrasive particle size, the slurry viscosity in the polishing area, the slurry flow rate, and material hardness. The material removal depth of GC-STP is about 1 μm. A smooth monocrystalline silicon surface is ultimately obtained after GC-STP treatment (Figure 9d).

## 5. Conclusions

This research proposes a novel green chemical shear-thickening method for polishing monocrystalline silicon. A new type of green chemical shear-thickening polishing slurry is prepared, the formula of which includes the following: silica nanoparticles, alumina abrasives, sorbitol, plant ash, polyethylene glycol, and deionized water. GC-STP technology uses alumina as an abrasive to achieve efficient and ultra-low damage processing of monocrystalline silicon wafers. The MRR achieved with GC-STP in this study was 4.568 μmh^−1^. After the GC-STP process, the surface roughness (Ra) was reduced to 0.278 nm, which is suitable for semiconductor monocrystalline silicon wafers. The principle of GC-STP was elucidated by scanning electron microscopy and FTIR spectroscopy to be as follows: First, hydroxyl ions are released from the plant ash solution to form an alkaline GC-STP slurry. In this alkaline environment, the surface of the monocrystalline silicon forms Si(OH)_2_^2+^ ions under the action of hydroxyl ions, and Si(OH)_2_^2+^ ions chelate with sorbitol in the GC-STP solution to form an unstable intermediate Si(OH)_4_. This is then decomposed into SiO_2_(OH)_2_^2−^ to form a softening layer. Finally, the softening layer is removed to obtain a monocrystalline silicon wafer with an ultra-smooth surface.

## Data Availability

Data will be made available on request.
